# Magnetic susceptibility and grain size distribution as prospective tools for selective exploration and provenance study of iron sand deposits: A case study from Aceh, Indonesia

**DOI:** 10.1016/j.heliyon.2021.e08584

**Published:** 2021-12-09

**Authors:** Bijaksana Satria, Zakia Masrurah, Silvia Jannatul Fajar

**Affiliations:** Faculty of Mining and Petroleum Engineering, Institut Teknologi Bandung, Jalan Ganesha 10, Bandung 40132, Indonesia

**Keywords:** Iron sand, Magnetic susceptibility, Magnetic mineralogy, Grain size distribution, Provenance study, Aceh, Indonesia

## Abstract

Almost all of the iron sand found in Indonesia, from Sumatra to Papua, is sandy deposit. Despite its variety of minerals, iron sand is commonly mined for low economic uses such as building material. As iron sands from different localities might have different characteristics (grain-size distribution, mineralogy, magnetic properties), such characteristics might in turn be used for provenance study or for selective mining. This study aims to examine iron sand deposits from two geographically close but geologically different regions, Lampanah and Anoi Itam, and to test the grain size distributions and their relationship with magnetic susceptibility as well as Fe content. In both Lampanah and Anoi Itam, the sizes of iron sands were predominantly medium sand (MS) and fine sand (FS), but they differed in mass percentage (M%). Generally, magnetic susceptibility increases as grain size decreases. Fe content is also grain size dependent, with higher Fe content in finer sizes. The results imply that the combination of grain size distribution and magnetic mineral composition might be used not only as a provenance indicator for iron sand deposits, but also as a criterion for selective mining.

## 1 Introduction

Sandy deposits, particularly iron sands, are valued economically, as the minerals they contain can be used in various applications, from magnetite for steel manufacturing (Brathwaite et al., 2017) to ilmenite, rutile, and leucoxene (IRL) for titanium feedstock (Rozendaal et al., 2017) to adsorbents for removing arsenic from water (Panthi and Wareham, 2011). Not surprisingly, studies have identified the quantity and size of iron sand deposits (Lawton and Hochstein, 1993) as well as the mineralogy and chemistry of these sands (Abdel-Karim et al., 2016; Abdel-Karim and Barakat, 2017; Ali et al., 2018; Tiwow et al., 2018). The provenance of iron sands has also been studied (Brathwaite et al., 2017; Ali et al., 2018) through their mineral compositions using various techniques (electron microprobe, x-ray fluorescence, x-ray diffraction, and scanning electron microscopy–energy-dispersive x-ray spectroscopy).

**Figure 1:**
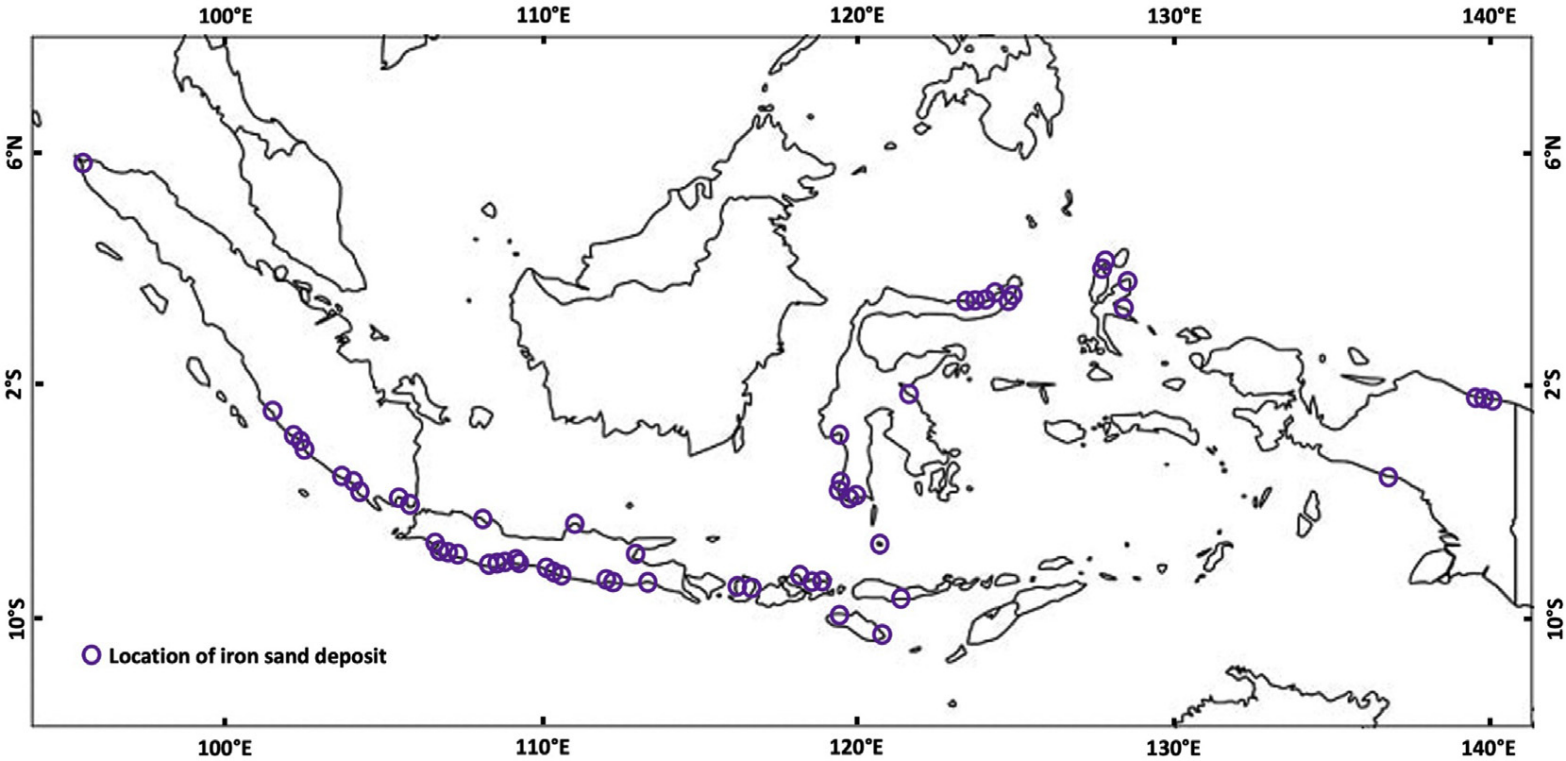
Distribution iron sand deposits in Indonesia (data from Kurnio (2007) and Rochani et al. (2008)).

As shown in Figure 1, Indonesia, with its unique geology, is home to iron sand deposits from Aceh, in the Northern tip of Sumatra, to Sarmi, on the Northern coast of Papua. The balance of mineral resources report indicates that iron sand resources amount to more than 4 billion tons, while iron sand reserves are 897 million tons (see Rochani et al., 2008). The proximity of the iron sand deposits to active volcanoes suggests that most of these deposits on volcanic islands such as Sumatra, Java, Bali, Lesser Sunda Islands, and Moluccas were derived as recent products of volcanic eruptions, although such deposits in Sulawesi and Papua were derived from the erosion of much older rocks (Kurnio, 2007). Some of these deposits are mined for low economic uses such as building materials and fillers for the cement industry. This type of mining is carried out by strip mining. However, some studies have been conducted to explore the possible uses of minerals in Indonesian iron sands as pigments (Mufti et al., 2014), heavy metal removers (Sari et al., 2017), magnetic fluids (Taufiq et al., 2017), and even magnetic sensors (Puspitaningrum et al., 2017). These types of applications might require selective mining, in which only selective minerals or grain sizes are exploited.

In this paper, we report the results of our study on the grain size distribution, magnetic susceptibility, and mineralogy of two distinct, yet geographically close, iron sand deposits in Aceh, Indonesia. This study is a maiden attempt to test whether different iron sand deposits have different grain size distributions and to verify whether different grain sizes indeed have different mineral compositions. If successful, the combination of grain size distribution and magnetic mineral composition might be used not only as a provenance indicator for iron sand deposits but also as a criterion for selective mining.

## 2 Materials and methods

**Figure 2:**
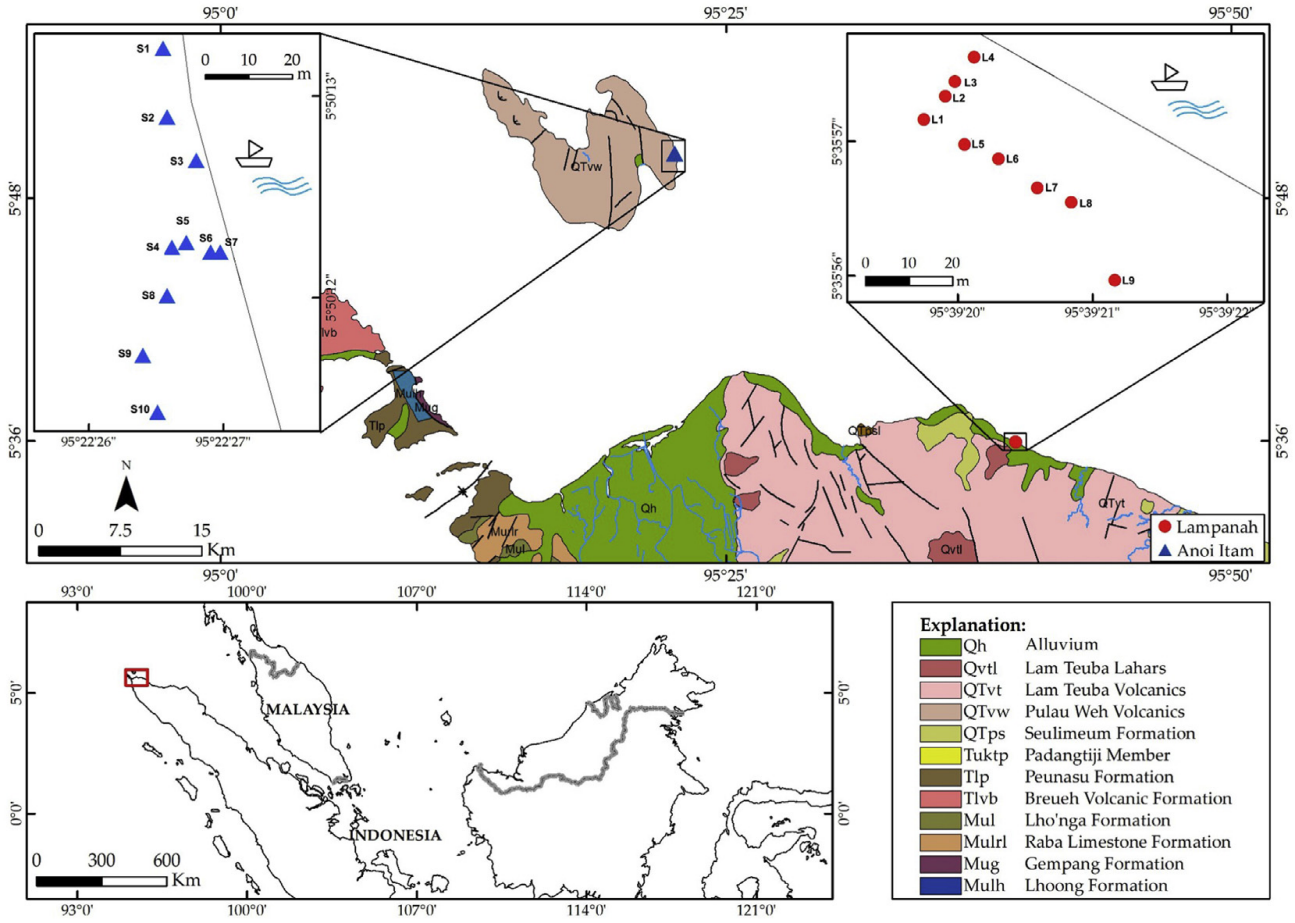
Map of study area and sample points marked with blue triangles in Lampanah and red circles in Anoi Itam. Geological information was obtained from Bennett et al. (1981).

Samples were taken from 2 sites, Lampanah and Anoi Itam beaches. Lampanah is located in the Northern tip of Sumatra (5° 35′ 57.2″ N; 95° 39′ 19.8″) to the east of Banda Aceh (the provincial capital of Aceh Darussalam), and Anoi Itam is located on Weh Island (5° 50′ 13.3″ N; 95° 22’ 27.1”) across a narrow strait to the north of Banda Aceh (see Figure 2). Both Lampanah and Anoi Itam beaches are not protected areas and permissions were obtained from local governments to collect samples from these two beaches. Lampanah iron sand deposits were derived from Quaternary Lam Teuba volcanic formation, while the Anoi Itam sand deposits were derived from Pulau Weh volcanic formation. Both Lam Teuba and Pulau Weh formations are characterized by basaltic andesitic to dacite lavas (Bennett et al., 1981). Due to their geographical conditions, these 2 beaches were not affected by the 2004 tsunami; thus, no tsunami deposits were found on these beaches. Nine iron sand samples (L-1 to L-9) were taken from Lampanah along the beach a few meters from each other (see Figure 2), and 10 samples (S-1 to S-10) were taken from Anoi Itam in similar fashion. These samples were collected between 5 and 11 January 2018. Each sample weighed about 5–6 kg. The samples were rinsed in running water and air dried in the laboratory for 14 days.

Each sample was then sieved through a 10-mesh sieve (2 mm opening) to eliminate particles larger than sand size. A small amount was set aside and is referred to as the bulk subsample. The remaining samples were then sieved through a series of 18-mesh, 35-mesh, 60-mesh, and 120-mesh sieves. Through this series of sieves, iron sand samples could be divided into 5 subsamples depending on their grain size, i.e., very coarse sand (VCS; particles passed through a 10-mesh sieve), coarse sand (CS; particles passed through an 18-mesh sieve), medium sand (MS; particles passed through a 35-mesh sieve), fine sand (FS; particles passed through a 60-mesh sieve), and very fine sand (VFS; particles passed through a 120-mesh sieve) (see Wenworth, 1922). The subsamples were then weighed using a digital scale to determine their weight percentage (M%), by dividing their weight by the total weight before sieving. Thus, in total there were 54 subsamples from Lampanah and 60 subsamples from Anoi Itam. Subsamples were designated by identification numbers in the form X-Y-ZZZ, where X is either L for Lampanah or S for Anoi Itam, Y is the sample number in a particular location (1–9 for Lampanah and 1 to 10 for Anoi Itam), and ZZZ indicates bulk (B), very coarse sand (VCS), coarse sand (CS), medium sand (MS), fine sand (FS), or very fine sand (VFS).

For each subsample (including the bulk samples), about 30 cm^3^ was set aside and placed inside 3 cylindrical sample holders (25.4 mm in diameter, 22 mm in height, and about 10 cm3 in volume) for magnetic susceptibility measurements. These samples in cylindrical holders were weighed using an Ohaus analytical balance before they were measured using a Bartington MS2 magnetic susceptibility system (Bartington Instruments Ltd., Witney, UK) with MS2B dual-frequency sensor. The results of these measurements were low-frequency (460 Hz) and high-frequency (4.6 kHz) mass-specific magnetic susceptibilities, denoted respectively as χ_LF_ and χ_HF_. The third parameter, termed frequency dependent susceptibility or susceptibility χ_FD (%)_, could be derived as χ_FD(%)_ = 100% × (χ_LF_ − χ_HF_)/χ_LF_.

Selected subsamples were also subjected to x-ray fluorescence (XRF) analysis using a Supermini 200 x-ray fluorescence spectrometer (Rigaku Corp., Tokyo, Japan) to identify their major and trace elements. Selected samples were also subjected to x-ray diffraction (XRD) analysis using a SmartLab x-ray diffractometer (Rigaku Corp., Tokyo, Japan) equipped with a Cu target to identify the minerals contained in the subsamples. For these analyses, a few grams of subsamples were pulverized so that they could pass through a 200-mesh sieve.

## 3 Results

**Figure 3:**
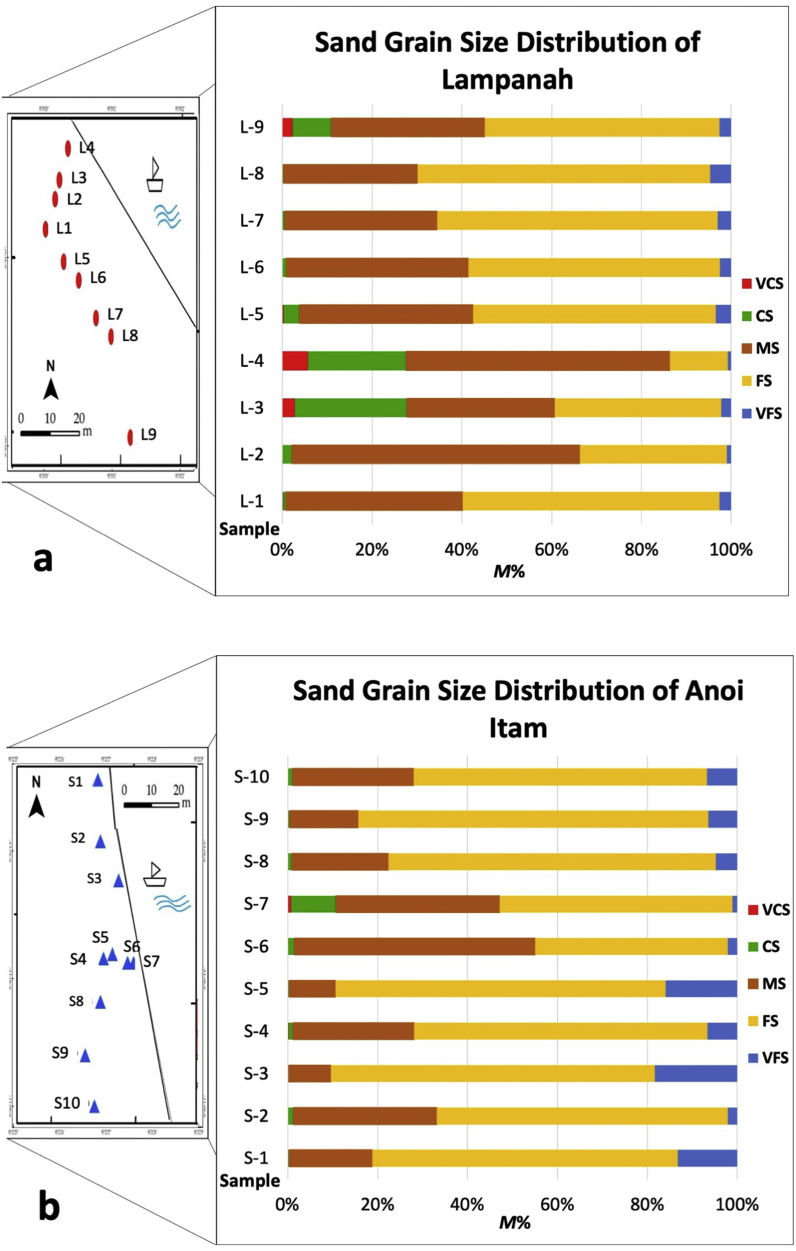
Results of sand grain size distribution of (a) Lampanah and (b) Anoi Itam.

**Figure 4:**
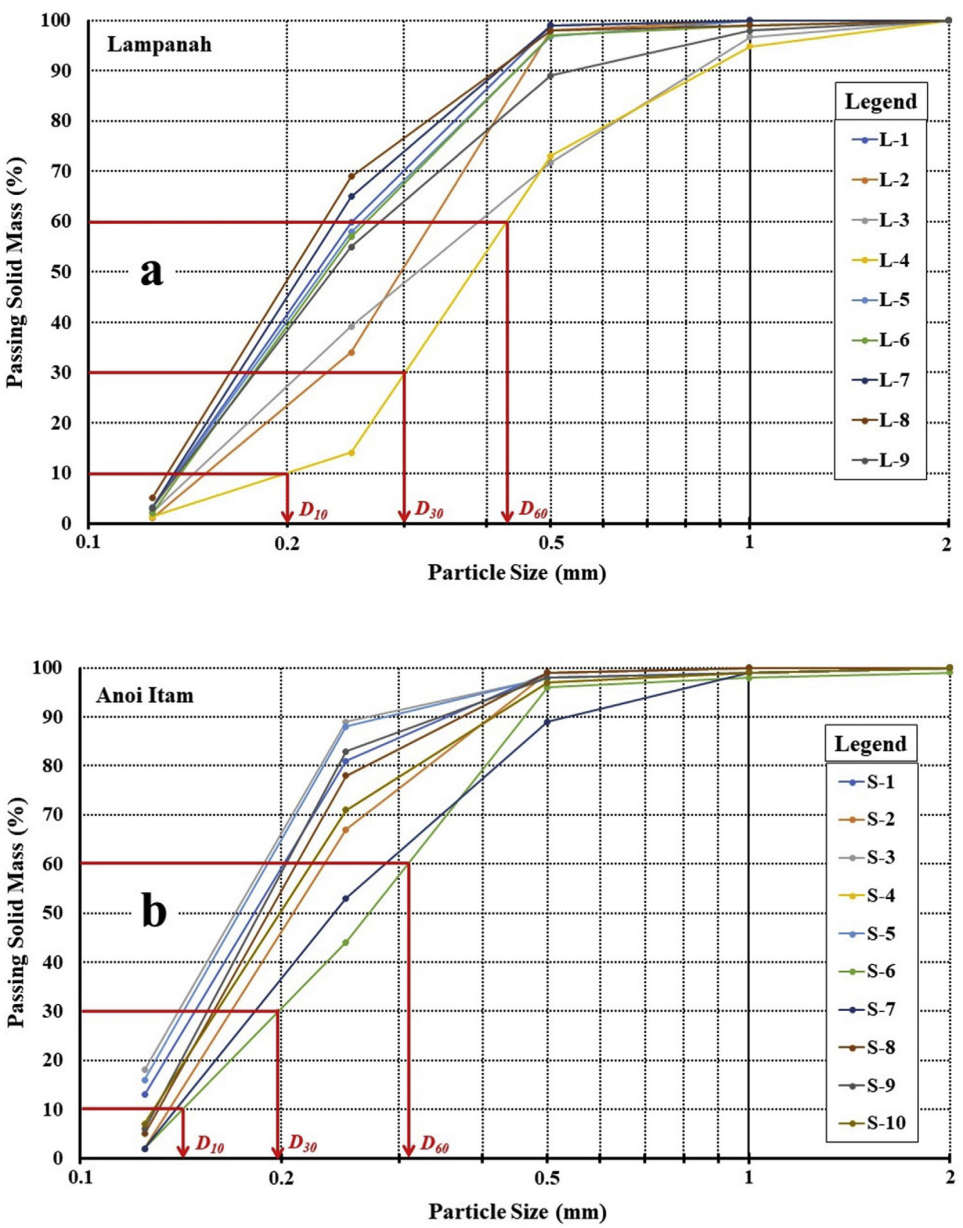
Particles size distribution for samples from Lampanah (a) and Anoi Itam (b). Red lines and red texts illustrate how *D*_*10*_, *D*_*30*_, and *D*_*60*_ were determined for samples L-4 and S-6. See text for further explanation.

Results of M% and magnetic susceptibility (χLF) for all subsamples from Lampanah and Anoi Itam are summarized in Table 1. This table shows that M% distribution differed for all subsamples. In both locations, the iron sand samples were mostly MS and FS sizes, and the other grain sizes (VCS, CS, and VFS) were very low in M%. In Lampanah, the average M% for MS and FS was 39% and 46%, respectively, while in Anoi Itam it was 25% and 65%, respectively. Results from Table 1 are also presented as graphs in Figures 3 and 4. There is no observable pattern in the distribution of grain sizes with relative distance to the sea (see Figure 3).

**Table 1:** *M%* and magnetic susceptibility (χ_LF_) in (× 10^−8^ m^3^/kg) for all iron sand subsamples from Lampanah (Site 1) and Anoi Itam (Site 2).

Sampling Point		Bulk		VCS		CS		MS		FS		VFS	
		*M%*	χ_LF_	*M%*	χ_LF_	*M%*	χ_LF_	*M%*	χ_LF_	*M%*	χ_LF_	*M%*	χ_LF_
Site 1	L-1	100%	4170.3 ± 1.9	1% <	851.4 ± 5.6	1%	1603.2 ± 1.3	38%	3753.9 ± 2.3	55%	3507.4 ± 3.2	3%	5453.0 ± 2.7
	L-2	100%	2904.0 ± 1.2	1% <	365.0 ± 8.1	2%	1297.6 ± 0.9	62%	1818.1 ± 2.0	32%	1286.5 ± 1.5	1%	6237.5 ± 3.9
	L-3	100%	729.1 ± 1.0	3%	861.9 ± 1.0	23%	710.9 ± 1.0	30%	740.8 ± 0.8	34%	684.8 ± 0.9	2%	3112.6 ± 2.2
	L-4	100%	677.1 ± 0.8	4%	860.7 ± 1.5	17%	785.0 ± 1.2	46%	736.6 ± 1.2	10%	886.1 ± 0.9	1%	4065.7 ± 1.1
	L-5	100%	2781.2 ± 2.3	1% <	719.6 ± 7.5	3%	930.9 ± 0.9	37%	3114.9 ± 1.9	52%	3536.6 ± 2.8	3%	2911.7 ± 3.3
	L-6	100%	3136.2 ± 2.6	1% <	699.6 ± 4.3	1%	1664.4 ± 1.0	40%	3221.8 ± 3.8	55%	3070.9 ± 3.9	2%	3472.1 ± 4.3
	L-7	100%	4090.3 ± 2.3	1% <	894.7 ± 4.5	1% <	2116.4 ± 1.0	34%	3868.7 ± 1.7	62%	4894.0 ± 2.1	3%	3111.4 ± 3.4
	L-8	100%	3189.0 ± 2.2	1% <	655.6 ± 5.1	1% <	1837.7 ± 1.9	29%	4627.0 ± 3.6	64%	3343.4 ± 3.6	5%	3639.5 ± 3.2
	L-9	100%	2037.8 ± 3.5	2%	930.1 ± 0.8	9%	874.0 ± 0.9	34%	1362.8 ± 1.1	52%	1569.7 ± 2.5	3%	3401.4 ± 1.3
	**Average**	**100%**	**2999.4 ± 2.0**	**1%**	**719.2 ± 4.3**	**6.2%**	**1313.3 ± 1.1**	**39%**	**2582.8 ± 2.0**	**46%**	**2531.0 ± 2.4**	**2%**	**3933.9 ± 2.8**
Site 2	S-1	100%	1628.3 ± 3.6	1% <	1260.6 ± 2.1	1% <	1883.7 ± 1.0	18%	4921.0 ± 1.8	68%	2752.1 ± 3.4	13%	4494.0 ± 1.6
	S-2	100%	3790.6 ± 2.1	1% <	587.6 ± 6.2	1%	1777.9 ± 1.6	32%	2907.1 ± 2.8	65%	2973.7 ± 6.1	2%	5028.0 ± 5.8
	S-3	100%	2928.2 ± 2.4	1% <	2319.9 ± 7.4	1% <	2127.0 ± 5.5	9%	4624.9 ± 3.1	71%	3171.3 ± 4.5	18%	3236.0 ± 3.9
	S-4	100%	5068.5 ± 3.2	1% <	3522.5 ± 3.1	1%	1398.7 ± 1.0	26%	5142.1 ± 5.2	64%	2940.5 ± 4.1	7%	4638.0 ± 6.0
	S-5	100%	3248.6 ± 4.1	1% <	1614.2 ± 4.1	1% <	1061.0 ± 5.2	10%	1973.8 ± 5.3	72%	3642.1 ± 2.8	16%	3552.0 ± 4.4
	S-6	100%	2346.8 ± 3.8	1% <	780.3 ± 4.0	1%	2474.9 ± 2.1	52%	3183.1 ± 4.4	42%	3941.6 ± 5.5	2%	887.7 ± 4.1
	S-7	100%	2114.2 ± 4.1	1%	844.9 ± 0.8	10%	1694.6 ± 0.3	36%	4267.0 ± 3.3	51%	3415.8 ± 6.6	1%	1102.5 ± 7.6
	S-8	100%	2934.2 ± 4.9	1% <	556.2 ± 5.0	1%	1962.8 ± 1.8	21%	2961.1 ± 2.7	73%	3075.9 ± 2.7	5%	3161.1 ± 7.3
	S-9	100%	2943.3 ± 4.8	1% <	2485.2 ± 4.3	1% <	2503.1 ± 6.7	15%	4267.3 ± 2.5	77%	2490.4 ± 2.4	6%	2367.2 ± 5.7
	S-10	100%	2004.4 ± 4.3	1% <	NA-	1% <	3486.3 ± 2.8	26%	1470.6 ± 2.7	64%	1933.5 ± 3.9	7%	2404.2 ± 4.2
	**Average**	100%	**2834.2 ± 3.7**	**0.1%**	**966.9 ± 3.7**	**2%**	**1897.3 ± 2.8**	**25%**	**3571.8 ± 3.4**	**65%**	**3033.7 ± 4.2**	**8%**	**3087.1 ± 5.1**

VCS, very coarse sand; CS, coarse sand; MS, medium sand; FS, fine sand; VFS, very fine sand.

Figure 4 shows the particle size distribution for all samples from Lampanah and Anoi Itam. The coefficient of uniformity (*C*_*U*_) and the coefficient of curvature (*C*_*C*_) of each subsample could easily be calculated from the curves in Figure 4. These two coefficients are defined respectively as *C*_*U*_ = *D*_60_/*D*_*10*_ and *C*_*C*_ = (*D*_*30*_ × *D*_*30*_)/(*D*_*60*_ × *D*_*10*_) where *D*_*10*_ is the particle size at which 10% of the particles are finer and 90% of the particles are coarser than *D*_*10*_ size, *D*_*30*_ is the particle size at which 30% of the particles are finer and 70% of the particles are coarser than *D*_*30*_ size and *D*_*60*_ is the particle size at which 60% of the particles are finer and 40% of the particles are coarser than *D*_*60*_ size (Chapuis, 2021). The values of *C*_*U*_ and *C*_*C*_ for Lampanah varies from 0.1833 and 0.970 (for subsample L-8) to 2.050 and 1.098 (for subsample L-4). For Anoi Itam subsamples, the values of *C*_*U*_ and *C*_*C*_ varies from 1.727 and 0.938 (for subsample S-3) to 2.384 and 0.993 (for subsample S-6). Despite slight differences in their values of *C*_*U*_ and *C*_*C*_, both Lampanah and Anoi Itam samples could be classified as poorly graded as expected for beach sand.

Table 1 also shows that the χ_LF_ value varied for each sand grain size. Based on the average, the χ_LF_ values for Lampanah sand grains increase from coarser to finest grain size, aligned as VCS = 719.2 ± 4.3 (× 10^−8^ m^3^/kg), CS = 1313.3 ± 1.1 (× 10^−8^ m^3^/kg), MS = 2582.8 ± 2.0 (× 10^−8^ m^3^/kg), FS = 2531.0 ± 2.0 (× 10^−8^ m^3^/kg), and VFS = 3933.9 ± 2.8 (× 10^−8^ m^3^/kg), with the highest χ_LF_ value being VFS size. Similar to Lampanah sand grain size, the χ_LF_ values for Anoi Itam sand grains also increase from coarser to finest grain size, aligned as VCS = 966.9 ± 3.7 (× 10^−8^ m^3^/kg), CS = 1897.3 ± 2.8 (× 10^−8^ m^3^/kg), MS = 3571.8 ± 3.4 (× 10^−8^ m^3^/kg), FS = 3033.7 ± 4.2 (× 10^−8^ m^3^/kg), and VFS = 3087.1 ± 5.1 (× 10^−8^ m^3^/kg), with the highest χ_LF_ value being MS size.

Samples from L-7 and S1 were selected for XRF analysis, and the results are shown in Table 2. The Fe content significantly increases from coarser sizes to finer sizes, while the Si and Ca contents decrease. The contents of Fe, Si, and Ca vary greatly in bulk samples from Lampanah (represented by L-7) and from Anoi Itam (represented by S-1). Fe content is significantly lower in the bulk sample of L-7 (45.2%) compared to S-1 (65.4%). The lower Fe content in L-7 is compensated by higher content of Si and Ca. Thus, the Fe, Si, and Ca contents in bulk samples might serve as a fingerprint for provenance study. The other elements (Na, Mg, Al, P, S, Cl, K, Ti, Cr, Mn, Ni, Zn, Zr, Sr, Rh, Rb, Ag, and V) are in much lower concentration and vary insignificantly in grain size.

**Table 2:** Results of XRF analysis of selected samples from Lampanah (L-7) and Anoi Itam (S-1). The chemical components mentioned and discussed specifically in the text (Si, Ca, and Fe) are shown in bold.

Sample	Chemical Component (mass%)																				
	Na	Mg	Al	Si	P	S	Cl	K	Ca	Ti	Cr	Mn	Fe	Ni	Zn	Zr	Sr	Rh	Rb	Ag	V
L-7 VFS	0.93	2.28	4.37	**12.00**	0.23	0.15	0.88	0.42	**2.98**	7.29	0.64	0.71	**66.90**	ND	0.08	0.23	ND	ND	ND	ND	ND
L-7 FS	1.10	4.78	4.88	**19.30**	0.37	0.07	0.86	0.45	**7.30**	5.68	0.14	0.74	**54.20**	ND	0.08	0.05	ND	ND	ND	ND	ND
L-7 MS	0.75	5.76	4.44	**21.10**	0.43	0.05	0.68	0.35	**7.77**	5.08	0.12	0.86	**52.40**	ND	0.08	0.04	0.03	ND	ND	ND	ND
L-7 CS	1.69	3.43	10.40	**42.40**	0.27	0.10	0.84	1.89	**16.10**	1.79	ND	0.55	**20.30**	ND	0.03	0.02	0.11	ND	0.03	0.08	ND
L-7 VCS	2.22	1.97	12.70	**44.00**	0.22	0.15	0.88	3.02	**18.60**	1.25	ND	0.33	**14.00**	ND	0.04	0.03	0.16	0.45	ND	ND	ND
L-7 B	0.96	4.99	6.38	**25.60**	0.37	0.06	0.49	0.66	**9.61**	4.63	0.11	0.79	**45.20**	0.03	0.06	0.06	ND	ND	ND	ND	ND
S-1 VFS	0.24	1.04	2.79	**4.36**	0.23	0.04	0.29	0.19	**0.91**	6.19	ND	0.62	**82.50**	ND	0.13	0.06	ND	ND	ND	ND	0.4
S-1 FS	0.23	2.32	2.53	**6.57**	0.32	0.03	0.29	0.17	**1.36**	6.46	ND	0.74	**78.50**	ND	0.10	ND	ND	ND	ND	ND	0.4
S-1 MS	0.54	8.65	4.66	**29.80**	0.34	0.03	0.11	0.43	**4.25**	2.64	ND	1.26	**47.10**	ND	0.09	ND	0.04	ND	ND	ND	ND
S-1 CS	1.26	4.97	10.30	**44.70**	0.22	0.10	0.10	2.57	**8.01**	0.85	ND	1.04	**25.60**	ND	0.07	0.02	0.09	ND	0.03	ND	ND
S-1 VCS	1.65	1.69	12.40	**47.20**	0.16	0.24	0.43	4.53	**15.00**	1.37	ND	0.31	**14.70**	ND	0.08	0.02	0.15	ND	0.06	ND	ND
S-1 B	0.55	4.24	4.14	**15.20**	0.29	0.03	0.53	0.34	**2.84**	4.95	ND	0.79	**65.40**	ND	0.09	0.03	ND	0.34	ND	ND	0.3

ND, not detected or less than 0.01%.

**Figure 5:**
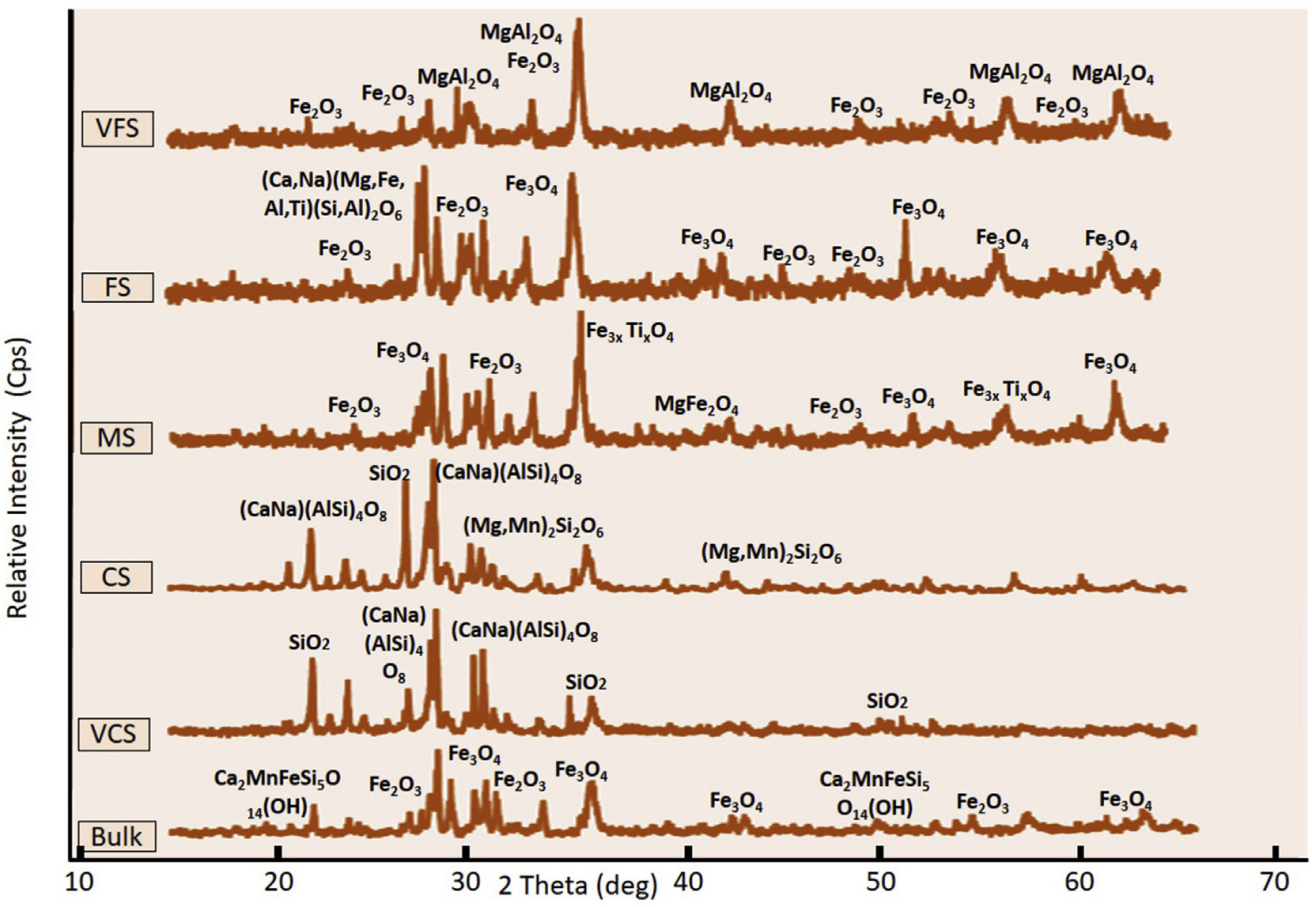
X-ray diffractograms of Lampanah samples (L-7).

**Figure 6:**
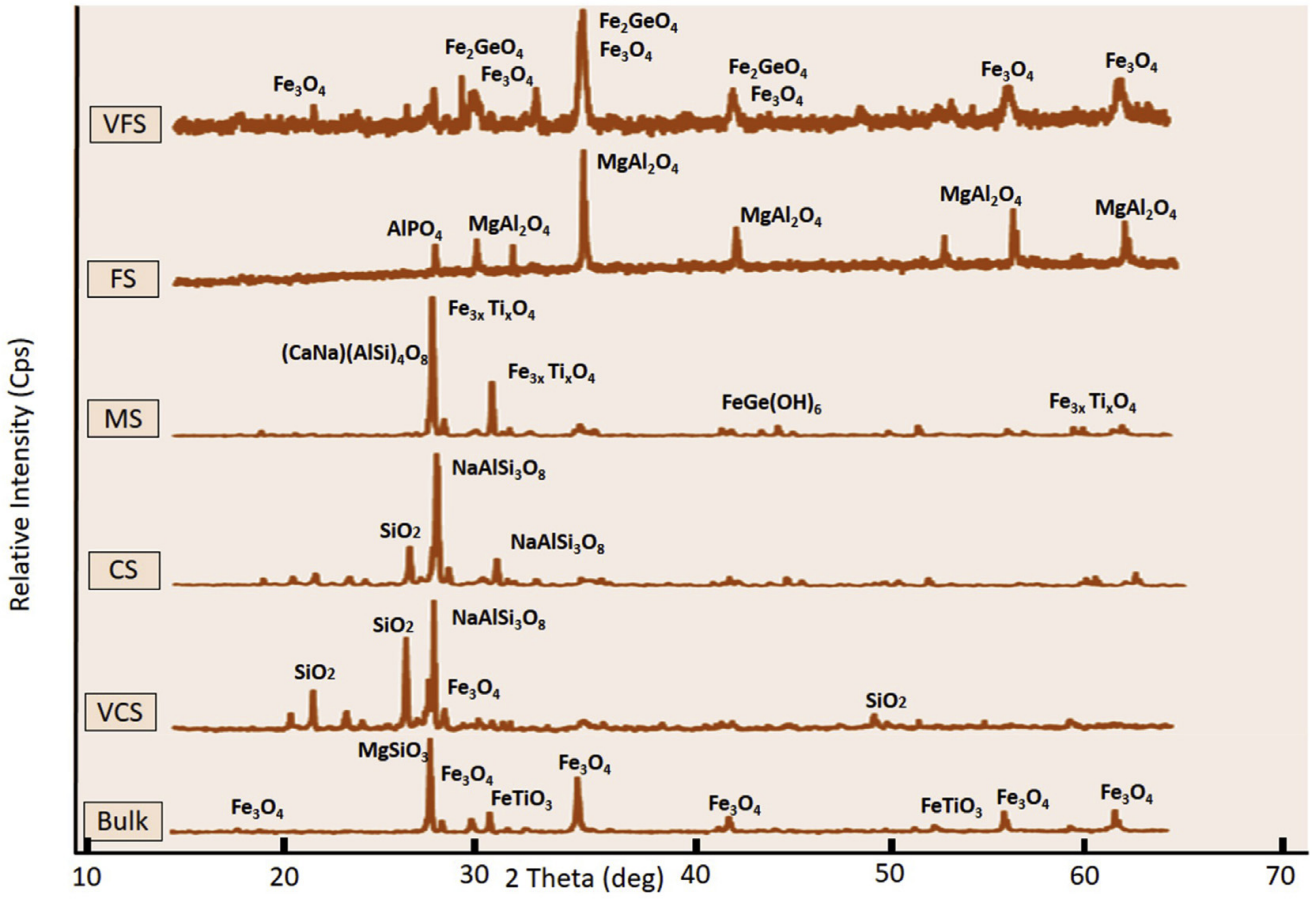
X-ray diffractograms of Anoi Itam samples (S-1).

Figures 5 and 6 show the combined diffractograms for selected samples L-7 (representing Lampanah) and S-1 (representing Anoi Itam), respectively. The bulk sample of L-7 shows a different pattern than that of the bulk sample of S-1, implying that their mineral contents are different and different mineralogy in iron sand might serve as a fingerprint for provenance study. As shown in Figures 5 and 6 and listed in Table 3, detected minerals in XRD analysis also differ from one grain size to another. Table 3 shows that for L-7 and S-1 samples, the minerals contained in the subsamples (VCS, CS, MS, FS, and VFS) differ from each other and from those of respective bulk samples. As expected, VFS samples are predominantly magnetite. However, a rare magnetite-like mineral called brunogeierite (Fe_2_GeO_4_) was also found in the VFS sample of S-1.

**Table 3:** Minerals identified from XRD diffractograms of Lampanah (L-7) and Anoi Itam (S-1) samples.

Grain Size	L-7 Samples	S-1 Samples
VFS	Spinel (MgAl_2_O_4_) Magnetite (Fe_3_O_4_) Hematite (Fe_2_O_3_)	Brunogeierite (Fe_2_GeO_4_) Magnetite (Fe_3_O_4_)
FS	Magnetite (Fe_3_O_4_) Hematite (Fe_2_O_3_) Augite ((CaNa) (MgFeAlTi) (Si Al)_2_ O_6_)	Spinel (MgAl_2_O_4_) Berlinite (AlPO_4_)
MS	Magnetite (Fe_3_O_4_) Hematite (Fe_2_O_3_) Titanomagnetite (Fe_3x_Ti_x_O_4_) Magnesioferrite (MgFe_2_O_4_)	Titanomagnetite (Fe_3x_Ti_x_O_4_) Andesine ((CaNa) (AlSi)_4_ O_8_) Sottite (FeGe(OH)_6_)
CS	Andesine ((Ca, Na) (Al, Si)_4_O_8_) Quartz (SiO_2_) Kanoite ((Mg Mn)_2_ Si_2_O_6_)	Quartz (SiO_2_) Albite (NaAlSi_3_O_8_)
VCS	Andesine ((CaNa) (AlSi)_4_ O_8_) Quartz (SiO_2_)	Quartz (SiO_2_) Albite (NaAlSi_3_O_8_) Magnetite (Fe_3_O_4_)
Bulk	Magnetite (Fe_3_O_4_) Hematite (Fe_2_O_3_) Andesine ((CaNa) (AlSi)_4_ O_8_) Manganbabingtonite (FeGe (OH)_6_)	Proto-enstatite (MgSiO_3_) Magnetite (Fe_3_O_4_) Ilmenite (FeTiO_3_)

## 4 Discussion

In densely populated countries such as Indonesia, massive exploitation of iron sand deposits for low economic use is a controversial issue, as it affects not only the environment but also the livelihoods of coastal communities. Therefore, selective and low-quantity exploitation might be preferable compared to the more common method of strip mining. As shown in this study, the Fe content varies greatly between localities as well as between different grain sizes, so combined grain-size distribution and magnetic susceptibility tests might be useful for preliminary investigation for selective mining. In Japan, iron sand is still being used (albeit in small quantities) in the making of classical weapons (Tanii et al., 2014).

In this study, although Fe content correlates linearly with particle size (finer particles tend to have higher Fe content), it does not always correlate linearly with magnetic susceptibility as some iron-bearing minerals could be paramagnetic (see Rosenbaum and Brownfield, 1999). Similarly, other non-iron minerals that include Ni, Co, Pt and even rare-earth metals could also be ferromagnetic. Rosenbaum and Brownfield (1999) found that when occurred as major components in minerals, metals such as manganese, copper, chromium, niobium, and tantalum, may enhance the magnetic susceptibilities of some members of a mineral series. Moreover, finer particle sizes do not necessarily indicate higher magnetic susceptibility. In both Lampanah and Anoi Itam, MS particles are generally more magnetic than FS particles.

Thus, if the objective of exploration is to find magnetic minerals or iron-bearing minerals then according to Tables 1 and 2, finer particle sizes (MS, FS, and VFS) are more promising than coarser particle sizes (VCS and CS).

The differences in grain-size distribution and mineral content between different iron sand localities are expected, as the sources of these deposits are different (Carranza-Edwards et al., 2009; Liu and Yang; 2018). Thus, grain-size distribution and mineral content might be used for provenance study of iron sand deposits. A similar study using iron sand samples from Papua and Java is currently under way. Compared to earlier studies, the magnetic susceptibility values (χ_LF_) of bulk samples from Lampanah and Anoi Itam are comparable to those of iron sand from Papua, Indonesia (Togibasa et al., 2018) but lower than those from Java (Yulianto et al., 2003). The results of the aforementioned studies, especially on the limits of magnetic susceptibility values, are expected to support the findings of this study.

Studies on iron sand deposits vary greatly in methodology, so it is not easy to compare characteristics of one deposit with another. Therefore, the authors of this study propose the following methods for iron sand characterization. First, iron sand samples are sieved (as in this study) so that their grain size distributions are known. Second, bulk and subsamples are measured for their magnetic susceptibility. Third, bulk and subsamples are measured for Fe, Si, and Ca content. Measurements of other elements and other types of analyses are optional.

The presence of brunogeierite (Fe_2_GeO_4_) in the VFS sample of S-1 is also interesting. This mineral is one of the compounds containing an oxyanion of germanium, collectively called germanates (Welch et al., 2001). Germanates could be used as analogues for silicates in studying mantle materials. The presence of brunogeierite in iron sand from Anoi Itam should be studied further, but at this time it is sufficient to mention that such presence might be used to differentiate one iron sand deposit from another.

## 5 Conclusions

Iron sand deposits from Lampanah and Anoi Itam in Aceh, Indonesia, were successfully characterized based on variations of their grain size, magnetic susceptibility, and mineralogy. These two deposits differ significantly in grain size distribution and magnetic mineralogy. Within the same locality, Fe, Si, and Ca contents vary depending on grain size. Fe content generally increases as grain size decreases, while Si and Ca contents increase as grain size increases. Although the bulk samples of the two iron sand deposits have similar magnetic susceptibility, the magnetic susceptibility of sieved subsamples vary with grain size. XRD analysis also shows that different grain sizes tend to have different mineralogy. The results imply that grain size distribution and magnetic mineralogy might be intrinsic characteristics that could be used for provenance study or as a basis for selective mining. Similar methods are being tested for iron sand deposits from other localities in Indonesia.

## Declarations

### Author contribution statement

Satria Bijaksana, Zakia Masrurah: Conceived and designed the experiments; Performed the experiments; Analyzed and interpreted the data; Wrote the paper.

Silvia Jannatul Fajar: Performed the experiments; Analyzed and interpreted the data; Contributed reagents, materials, analysis tools or data; Wrote the paper.

### Funding statement

This work was supported by Institut Teknologi Bandung through a P3MI research grant to Global Geophysics Research Group for fiscal year 2018 (56/SK/I1.C04/KP/2018).

### Data availability statement

Data will be made available on request.

### Declaration of interests statement

The authors declare no conflict of interest.

### Additional information

No additional information is available for this paper.
